# A Multifaceted Approach to Diabetic Foot Ulcer Healing Beyond HbA1c: A Prospective Observational Study in a Tertiary Care Hospital in Chennai

**DOI:** 10.7759/cureus.88136

**Published:** 2025-07-17

**Authors:** Mughilan Selvaraj, Santhaseelan R.G.

**Affiliations:** 1 General Surgery, Sree Balaji Medical College and Hospital, Chennai, IND

**Keywords:** albumin, chronic wounds, dfu predictors, diabetic foot ulcer, hba1c, inflammatory markers, ulcer classification, ulcer healing, wagner grade, wound healing

## Abstract

Background: Diabetic foot ulcers (DFUs) are among the most debilitating complications of diabetes mellitus, often leading to delayed healing, infections, and potential limb loss. While glycemic control, typically measured by HbA1c, has been a primary focus in DFU management, recent evidence suggests that healing outcomes are influenced by a broader range of factors.

Objective: To evaluate the predictive role of clinical, biochemical, and inflammatory parameters beyond HbA1c in the healing of DFUs.

Methodology: This longitudinal observational study included 220 patients with DFUs treated at a tertiary care hospital in Chennai. Data were collected on demographics, ulcer severity (Wagner Grade), glycemic status (HbA1c), biochemical markers (C-reactive protein [CRP], interleukin-6 [IL-6], tumor necrosis factor-alpha [TNF-α], and serum albumin), and clinical outcomes. Statistical analyses were performed using regression and correlation models to assess associations with time to ulcer healing.

Results: HbA1c showed a significant correlation with Wagner Grade but did not independently predict healing time. Serum albumin and Wagner Grade emerged as significant predictors of healing duration (*P* < 0.05). Although inflammatory markers such as CRP, IL-6, and TNF-α were elevated in many patients, they did not show a statistically significant correlation with healing outcomes.

Conclusions: DFU healing is a multifactorial process influenced by nutritional status and ulcer severity in addition to glycemic control. Integrating assessments such as Wagner Grade and serum albumin with traditional glycemic markers can enhance the prediction of healing outcomes and guide more effective management strategies.

## Introduction

Diabetic foot ulcers (DFUs) are a major complication of diabetes mellitus, contributing significantly to morbidity, hospitalizations, and lower limb amputations worldwide [1]. Recurrence rates are high, and despite advances in wound care, healing remains suboptimal in many cases [2]. The clinical outcome of DFUs depends on a complex interplay of factors, including glycemic control, infection, ulcer depth, vascular status, and patient compliance [3].

Traditionally, glycemic control has been considered the cornerstone of ulcer healing, often measured by HbA1c. However, recent studies suggest that HbA1c alone may not be a sufficient predictor of healing outcomes [4]. Infection, peripheral arterial disease, and immunocompromise are major risk factors for ulcer deterioration [5].

Treatment modalities for DFUs have advanced to include biologics such as Dermagraft and Graftskin, both of which have shown favorable healing outcomes in randomized clinical trials [6,7]. Vacuum-assisted closure (VAC) therapy has also demonstrated improved healing rates in difficult-to-treat ulcers [8]. The Wagner grading system remains widely accepted for classifying DFUs based on depth and infection extent [9].

Despite therapeutic advancements, guidelines and regulatory bodies continue to stress the need for robust, evidence-based strategies for chronic wound management [10]. Furthermore, multidisciplinary care and early intervention are key factors for reducing amputation rates and enhancing outcomes [11,12]. The effectiveness of offloading techniques and footwear, and the role of emerging pharmacologic agents, remain critical areas of ongoing research [13,14].

Finally, epidemiological data show that nontraumatic amputations in diabetic patients far exceed those in non-diabetics, underscoring the urgent need for optimized DFU care in endemic regions such as India [15].

## Materials and methods

This prospective longitudinal observational study was conducted over a one-year period from June 2022 to May 2023 in the Department of General Surgery at Sree Balaji Medical College and Hospital, Chennai. The objective was to evaluate the impact of glycemic control and additional clinical, biochemical, and inflammatory markers on the healing outcomes of DFUs. A total of 220 patients diagnosed with type 2 diabetes mellitus and presenting with DFUs were enrolled after obtaining written informed consent.

Inclusion criteria were: patients aged 18 years and above with type 2 diabetes mellitus, the presence of at least one clinically diagnosed DFU, and willingness to adhere to treatment and follow-up protocols.

Exclusion criteria included: patients with non-DFUs (e.g., venous, arterial, traumatic, or malignant ulcers), end-stage organ failure (cardiac, hepatic, or renal), terminal malignancy, known immunodeficiency, or those who were pregnant or lactating.

The sample size was calculated using the formula for estimating a proportion with a specified absolute precision: *n* = *Z*² × *p* × (1 - *p*)/*d*², where *Z* = 1.96 (for 95% confidence level), *p* = expected healing proportion (0.67), and *d* = absolute precision (0.07). Substituting into the formula: *n* = (1.96)² × 0.67 × (1 - 0.67) / (0.07)² ≈ 173. After accounting for an estimated 20% attrition rate, the final sample size was increased to 220. This calculation was performed using OpenEpi software, version 3.01.

Each patient underwent a thorough clinical evaluation, and ulcers were graded using the Wagner classification system. Ulcer surface area and depth were recorded. Biochemical investigations included HbA1c, serum albumin, C-reactive protein (CRP), interleukin-6 (IL-6), and tumor necrosis factor-alpha (TNF-α). Patients were monitored over three months, and ulcer healing was assessed based on complete epithelialization without discharge or infection. Data were entered into Microsoft Excel and analyzed using IBM SPSS Statistics for Windows, version 23.0 (IBM Corp., Armonk, NY). Continuous variables were expressed as mean ± standard deviation, and categorical variables as frequencies and percentages. The Chi-square test and Pearson correlation were used to assess associations, and logistic regression analysis was performed to identify independent predictors of healing. A *P*-value < 0.05 was considered statistically significant.

## Results

A total of 220 patients with type 2 diabetes mellitus and diabetic foot ulcers were enrolled. Of these, 114 (51.8%) were male and 106 (48.2%) were female. A history of smoking was present in 103 patients (46.8%), while 110 patients (50.0%) reported alcohol use. Comorbidities such as hypertension, coronary artery disease, or chronic kidney disease were documented in 166 patients (75.5%). Ulcer recurrence was seen in 115 patients (52.3%). At the end of the three-month follow-up, 115 patients (52.3%) achieved complete ulcer healing, while 105 (47.7%) had non-healed ulcers. The association of healing status with categorical variables such as sex, smoking, alcohol, comorbidities, and recurrence was assessed using the Chi-square test, as shown in Table 1.

**Table 1:** Association between clinical parameters and ulcer healing status. Ulcer recurrence was significantly associated with poor healing outcomes (*P* < 0.001). Other demographic and lifestyle factors were not significantly associated with healing.

Parameter	Ulcer healed (*n*)	Ulcer not healed (*n*)	*P*-value	Test used
Male	65	49	0.89	Chi-square test
Female	50	56		
Smoking history	52	51	0.76	Chi-square test
Alcohol use	58	52	0.91	Chi-square test
Presence of comorbidities	85	81	0.62	Chi-square test
Ulcer recurrence	38	77	<0.001	Chi-square test

Ulcers were graded using the Wagner classification system. Grade 1 ulcers were most frequent (68, 30.9%), followed by Grade 3 (54, 24.5%), Grade 2 (51, 23.2%), and Grade 4 (47, 21.4%). The Wagner grade showed a significant inverse association with healing status; lower grades healed better. Statistical significance was determined using the Chi-square test, as shown in Table 2.

**Table 2:** Distribution of Wagner grades and association with healing. A statistically significant association was observed between higher Wagner grades and poor healing outcomes (*P* < 0.001).

Wagner grade	Ulcer healed (*n*)	Ulcer not healed (*n*)	Total patients (*n*)	Percentage of total (%)	*P*-value	Statistical significance
Grade 1	47	21	68	30.9%		
Grade 2	27	24	51	23.2%		
Grade 3	21	33	54	24.5%		
Grade 4	20	27	47	21.4%		
Total	115	105	220	100%	<0.001	Significant

For continuous variables like HbA1c, CRP, IL-6, TNF-α, and albumin, mean values were compared between healed and non-healed groups using the Independent t-test. HbA1c and albumin were significantly associated with healing. Inflammatory markers (CRP, IL-6, and TNF-α), while elevated, did not show statistically significant differences as shown in Table 3.

**Table 3:** Biochemical parameters in healed vs. non-healed ulcers. Lower HbA1c and higher serum albumin levels were significantly associated with healing. Inflammatory markers were not statistically predictive.

Parameter	Healed (Mean ± SD)	Not healed (Mean ± SD)	*P*-value	Test used
HbA1c (%)	8.63 ± 1.67	9.44 ± 1.77	0.02	Independent t-test
CRP (mg/L)	17.01 ± 7.35	18.36 ± 7.72	0.19	Independent t-test
IL-6 (pg/mL)	42.31 ± 16.95	44.80 ± 17.18	0.26	Independent t-test
TNF-α (pg/mL)	12.69 ± 4.28	13.17 ± 4.56	0.38	Independent t-test
Serum albumin (g/dL)	3.35 ± 0.42	3.10 ± 0.42	0.01	Independent t-test

## Discussion

This study reinforces the notion that DFU healing is multifactorial and cannot be solely predicted by HbA1c levels. While HbA1c did show a statistically significant association with healing, several patients with good glycemic control still exhibited delayed or non-healing ulcers, a phenomenon supported by Armstrong et al., who emphasized the high recurrence and chronicity of DFUs despite controlled glucose levels [1].

The Wagner grading system was shown to be a strong predictor of healing outcomes. Patients with Wagner Grade 1 ulcers had the highest healing rate, while Grades 3 and 4 were associated with prolonged healing or treatment failure. This is in line with the observations made by Blume et al. and Prompers et al., who reported that deeper ulcers and those with ischemia or infection responded poorly to conservative treatments [2,3].

Inflammatory and nutritional markers such as CRP and albumin, though variable, were important adjuncts in assessing healing potential. However, as Jeffcoate et al. noted, these markers often lack specificity and should not be used in isolation [4].

Infection remains a critical determinant in healing outcomes. Lavery et al. identified infection as a principal driver for lower extremity hospitalization and amputation [5]. In our study, many non-healing ulcers were either infected or had recurrent presentations.

Therapeutic modalities, including Dermagraft and Graftskin, have shown improved healing outcomes in controlled settings [6,7]. However, cost and availability limit their widespread application in low-resource environments like ours. In such contexts, VAC therapy, described by Argenta and Morykwas, offers a practical and effective alternative, especially in post-debridement wounds [8].

The use of the Wagner classification system was validated in our cohort, supporting its clinical utility in resource-constrained settings [9]. Further, international guidelines continue to emphasize structured wound care approaches and product development to address chronic wounds [10]. Multidisciplinary team involvement remains crucial. Studies by Jeffcoate and Harding and by Game et al. have demonstrated that coordinated care significantly improves ulcer healing and reduces major amputation rates [11,12].

Offloading techniques and appropriate footwear, as reviewed by Bus et al., play a vital role in prevention and recurrence reduction [13]. Additionally, Lipsky’s work on diabetic foot infections highlights the importance of early and targeted antimicrobial therapy [14]. Finally, the high incidence of nontraumatic amputations in diabetic populations, as described by Calle-Pascual et al., should drive public health policies toward early screening and integrated DFU management (Table 4) [15].

**Table 4:** Summary of discussion points in relation to existing literature.

Topic	Key findings in this study	Supporting references
HbA1c vs. Healing	HbA1c significant, but not the sole, predictor	Armstrong et al. [1]
Wagner Grade	Strong inverse correlation with healing	Blume et al. [2], Wagner [9]
Infection and Recurrence	Poor healing is associated with recurrence/infection	Lavery et al. [5], Lipsky [14]
Dermagraft and Graftskin	Biologics are beneficial but costly	Marston et al. [6], Veves et al. [7]
VAC therapy	Effective post-debridement	Argenta et al. [8]
Role of multidisciplinary teams	Better healing and reduced amputations	Jeffcoate and Harding [11], Game et al. [12]
Epidemiology and amputation risk	High amputation in diabetics	Calle-Pascual et al. [15]

## Conclusions

Glycemic control, while essential, does not act in isolation in predicting DFU healing. Parameters such as ulcer grade and serum albumin offer valuable insights into healing potential. Future management protocols must incorporate a holistic, multidisciplinary approach, addressing glycemic control, nutritional support, wound care strategies, and early grading, to reduce amputation rates and promote timely healing in diabetic foot ulcer patients.
